# *PALM3* and hearing loss: a potential dual diagnosis interfering with novel gene discovery

**DOI:** 10.1038/s41431-026-02206-7

**Published:** 2026-07-29

**Authors:** Paria Najarzadeh Torbati, Lukas Hallbrucker, Michaela A. H. Hofrichter, Daniel Owrang, Jonas Setzke, Manfred W. Kilimann, Anahid Hemmatpour, Mohsen Rajati, Ehsan Ghayoor Karimiani, Thomas Haaf, Christian Vogl, Barbara Vona

**Affiliations:** 1 Department of Medical Genetics, Next Generation Genetic Polyclinic, Mashhad, Iran; 2 Department of Biology, Mashhad Branch, Islamic Azad University, Mashhad, Iran; 3 Auditory Neuroscience Group, Institute of Physiology, Medical University Innsbruck, Innsbruck, Austria; 4 Institute of Clinical Genetics and Genomic Medicine, University Hospital Würzburg, Würzburg, Germany; 5 Institute for Auditory Neuroscience and Inner Ear Lab, University Medical Center Göttingen, Göttingen, Germany; 6 Max-Planck-Institute for Multidisciplinary Sciences (City Campus), Göttingen, Germany; 7 Department of Otorhinolaryngology, School of Medicine, Ghaem Hospital, Sinus and Surgical Endoscopic Research Center, Mashhad University of Medical Sciences, Mashhad, Iran; 8 Centre for Neuromuscular Diseases, UCL Queen Square Institute of Neurology, London, UK; 9 Institute of Human Genetics, Julius-Maximilians-University Würzburg, Würzburg, Germany; 10 Auditory Neuroscience and Optogenetics Laboratory, German Primate Center, Göttingen, Germany; 11 Collaborative Research Center 1690 (CRC1690), University of Göttingen, Göttingen, Germany; 12 Department of Obstetrics and Gynecology, Brigham and Women’s Hospital, Harvard Medical School, Boston, MA USA; 13 Program in Medical and Population Genetics, Broad Institute of MIT and Harvard, Cambridge, MA USA

**Keywords:** Paediatric neurological disorders, Genetic counselling

## Abstract

Hereditary hearing loss is highly genetically heterogeneous, with emerging overlap between genes implicated in early-onset and age-related hearing loss. We report a consanguineous family with autosomal recessive, non-syndromic hearing loss in which the proband harbors a homozygous splice-site variant in *PALM3* NM_001145028.2:c.314+1G>A and a homozygous missense variant in *OTOA* NM_144672.4:c.1939G>C p.(Gly647Arg). A minigene assay for the *PALM3* variant demonstrated aberrant splicing with exon skipping, resulting in either a frameshift or a large in-frame deletion, both consistent with loss of function and impacting all known transcripts. While the organ of Corti from 12-month-old heterozygous *Palm3* mice showed preserved overall architecture, a study describing *Palm3* knockout mice exhibit auditory dysfunction, supporting a possible auditory phenotype with loss of function. Although a dual molecular diagnosis cannot be excluded, the combined genetic, functional, and comparative data support *PALM3* as a candidate gene potentially contributing to autosomal recessive hearing loss.

## Introduction

Cochlear hair cells convert mechanical sound waves into electrical signals. This process relies on ribbon synapses, electron-dense structures that tether synaptic vesicles to enable sustained neurotransmission [1]. In mammals, two functionally distinct hair cell types cooperate in auditory processing: inner hair cells (IHCs), which transmit acoustic signals via ribbon synapses, and outer hair cells (OHCs), which amplify cochlear motion through voltage-dependent electromotility [2]. Each IHC forms monosynaptic connections with multiple auditory nerve fibers, and synaptic heterogeneity contributes to diverse firing properties [3].

Hereditary hearing loss is highly genetically heterogeneous. More than 155 genes are associated with early-onset non-syndromic hearing loss, while genome-wide association studies have identified approximately 80 candidate genes for age-related hearing loss (ARHL), despite many causative genes still pending discovery [4]. Although ARHL genetics has not been used for routine diagnostics yet, previous studies on twins and families indicated that genetic factors play a key role, for approximately 50–70%, in ARHL [5,6]. These genetic regions highlight biological pathways that overlap with Mendelian deafness genes and suggest further in-depth studies are needed [7].

Paralemmins are a family of membrane proteins, PALM1-3, and Palmdelphin, which contribute to membrane shaping and cytoskeletal interactions [8,9]. A recent preprint study demonstrated that *Palm3* is expressed in both IHCs and OHCs and concentrates at their lateral membranes. *Palm3*-knockout (KO) mice exhibit early-onset, progressive hearing loss due to shortening of OHCs, membrane disorganization, and cytoskeletal disruption. Adeno-associated virus-mediated *Palm3* delivery partially restored structure and function [2].

Here, we report a family with autosomal recessive (AR) hearing loss harboring the first known biallelic loss-of-function (LOF) variant in *PALM3*. The affected individual also segregates a homozygous missense variant in the established deafness gene, *OTOA* (DFNB22), suggesting a potential dual diagnosis consistent with the extreme heterogeneity of hereditary hearing loss. Functional analysis demonstrates *PALM3* exon skipping, and phenotypic concordance with *Palm3*-KO mice supports a LOF mechanism. These findings support *PALM3* as a promising candidate human deafness gene involved in hair cell membrane resilience and cochlear function.

## Methods

All procedures describing patient recruitment, clinical assessment, exome sequencing, bioinformatics, variant classification, in vitro splice assay and the histological analysis of cochlear hair cells in *Palm3* heterozygous and wild-type mice are described in the Supplementary Materials.

## Results

### Case report

The proband is an adult woman born to consanguineous parents of Iranian descent who presented with bilateral, sensorineural hearing loss. No vestibular dysfunction, visual impairment, or other syndromic features were observed. Her father and brother were also affected. Although precise age-of-onset information could not be obtained despite repeated attempts to recontact the family, available clinical information indicated that hearing loss manifested well before the age typically associated with age-related hearing loss. The presence of multiple consanguineous marriages and three affected individuals within nuclear families suggested an AR inheritance pattern despite hearing loss being present in three generations. The proband is married to an unrelated hearing-impaired man.

### Genomic analysis

Whole-exome sequencing was performed for the proband and her spouse. The proband presented a homozygous splice-site variant in *PALM3* NM_001145028.2:c.314+1G>A and a homozygous variant in *OTOA* NM_144672.4:c.1939G>C p.(Gly647Arg) (Table 1). The *OTOA* variant has been previously published [10] and is classified as pathogenic in the Deafness Variation Database and as a variant of uncertain significance (VUS) in ClinVar through manual classification using the hearing loss adjusted ACMG guidelines. Her husband was identified with a homozygous variant in *TMC1* NM_138691.2:c.1535G>A p.(Arg512Gln). According to the American College of Medical Genetics and Genomics (ACMG) variant interpretation guidelines for genetic hearing loss [11], both variants in *OTOA* (PM2_Supporting, PP1_Supporting, PP3_Moderate) and *TMC1* (DFNB7/11) (PM2_Supporting) were classified as a VUS (Table 1). The *PALM3* NM_001145028.2:c.314+1G>A variant was absent in population frequency databases and predicted to cause abolishment of the native splice donor site unanimously by SpliceSiteFinder-like, MaxEntScan, NNSPLICE, GeneSplicer, SpliceAI, AbSplice, and varSEAK (Supplementary Fig. 1A). Although the variant affects the canonical +1 splice donor position and demonstrated aberrant splicing in vitro, a formal ACMG classification was not assigned because *PALM3* currently lacks an established human gene–disease relationship.

**Table 1:** Variants identified in the present study.

Gene (Transcript) chromosomal GRCh38	c. position	p. position	AF gnomAD (v.4.1.0)	MAF gnomAD (v4.1.0)	MAF Pop gnomAD (v4.1.0)	TOPMed v8	All of Us	SIFT	PP-2	FATHMM	MT	REVEL	CADD	ACMG Classification
*PALM3* (NM_001145028.2) NC_000019.10:g.14056661C>T	c.314+1G>A	p.?	NP	NP	NP	NP	NP	NS	NS	NS	NS	NS	35.0^1^	NA*
*OTOA* (NM_144672.4) NC_000016.10:g.21726581G>C	c.1939G>C	p.(Gly647Arg)	3.01e-6	1.7e-4	Middle Eastern	NP	NP	T	PrD	D	D	D	24.7^2^	VUS (PM2_Supporting, PP1_Supporting, PP3_Moderate)
*TMC1* (NM_138691.2) NC_000009.12:g.72792321G>A	c.1535G>A	p.(Arg512Gln)	5.0e-6	3.3e-5	Admixed American	4.0e-6	1.4e-5	T	PrD	D	D	U	33.0^1^	VUS (PM2_Supporting)

Pathogenicity is represented as ^1^deleterious or ^2^neutral prediction. PM2, rare in population databases; PP3, computational evidence supports deleterious effect. * ACMG classification was not assigned for the *PALM3* variant because this gene currently lacks an established human gene-disease relationship. The *PALM3*, *OTOA*, and *TMC1* variants have been submitted to ClinVar under accession numbers SUB16130109 and SUB16231678.

*AF* allele frequency, *B* benign, *chr* chromosome, *D* Deleterious, *F* family, *MAF* maximum allele frequency, *MT* MutationTaster, *NA* not applicable, *NP* not present, *NS* not scored, *Pop* population, *PP2* PolyPhen-2, *PrD* probably damaging, *T* tolerated, *U* Uncertain, *VUS* Variant of Uncertain Significance.

Segregation analysis by Sanger sequencing confirmed that the proband was homozygous for the *PALM3* and *OTOA* variants. Her affected father was homozygous for the *OTOA* variant and heterozygous for the *PALM3* variant. The unaffected mother was a heterozygous carrier of both *OTOA* and *PALM3* variants. Her affected brother was unavailable for testing. The proband’s husband was confirmed with *PALM3* and *OTOA* reference alleles and his hearing loss is suspected to be due to the homozygous *TMC1* variant, although both *OTOA* and *PALM3* variants are currently classified as VUS (Table 1).

### Functional studies

**Fig. 1:**
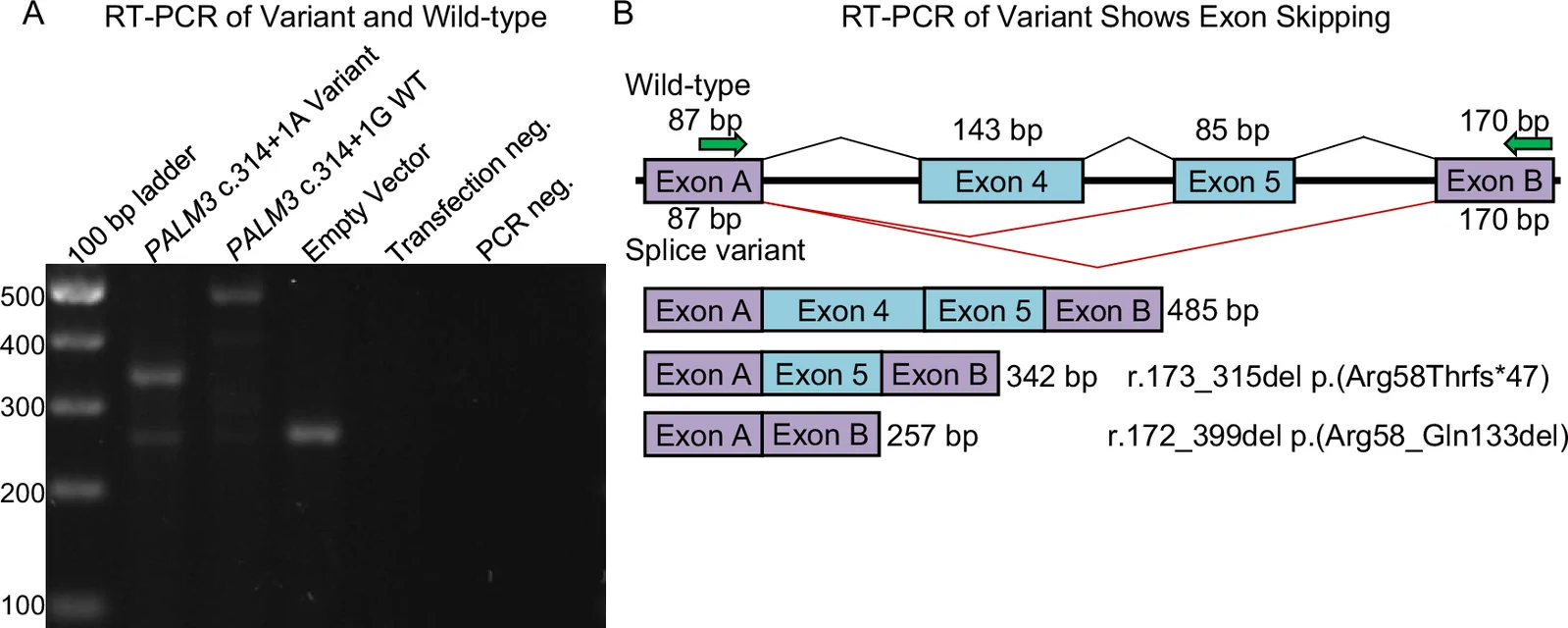
In vitro splicing assay. **A** RT-PCR of the in vitro splicing assay testing the *PALM3* NM_001145028.2:c.314+1G>A variant. The variant (c.314+1A) shows two bands corresponding to skipping of exon 4 and exons 4–5, while the wild-type (c.314+1G), empty vector, transfection and PCR controls performed as expected. **B** A schematic of the results of the in vitro splice assay. The upper panel shows the wild-type splicing result containing the expected exons 4–5 joined with black lines. The bottom panel shows the skipped exon splicing results. Skipped exon 4 and 4–5 are marked with red lines. The illustration below shows the splicing results from the agarose gel showing the RT-PCR with the amplicon sizes shown. The aberrant splicing results are shown with their r. and p. positions.

A minigene assay evaluated the effect of the *PALM3* NM_001145028.2:c.314+1G>A variant on splicing. RT-PCR demonstrated aberrant splicing consistent with exon skipping (Fig. 1A, B). Skipping of exon 4 results in a frameshift (r.173_315del, causing a frameshift p.(Arg58Thrfs*47)), while skipping of exons 4–5 would cause an in-frame deletion (r.172_399del p.(Arg58_Gln133del)). All known *PALM3* transcripts are predicted to be affected (Supplementary Fig. 1B).

**Fig. 2:**
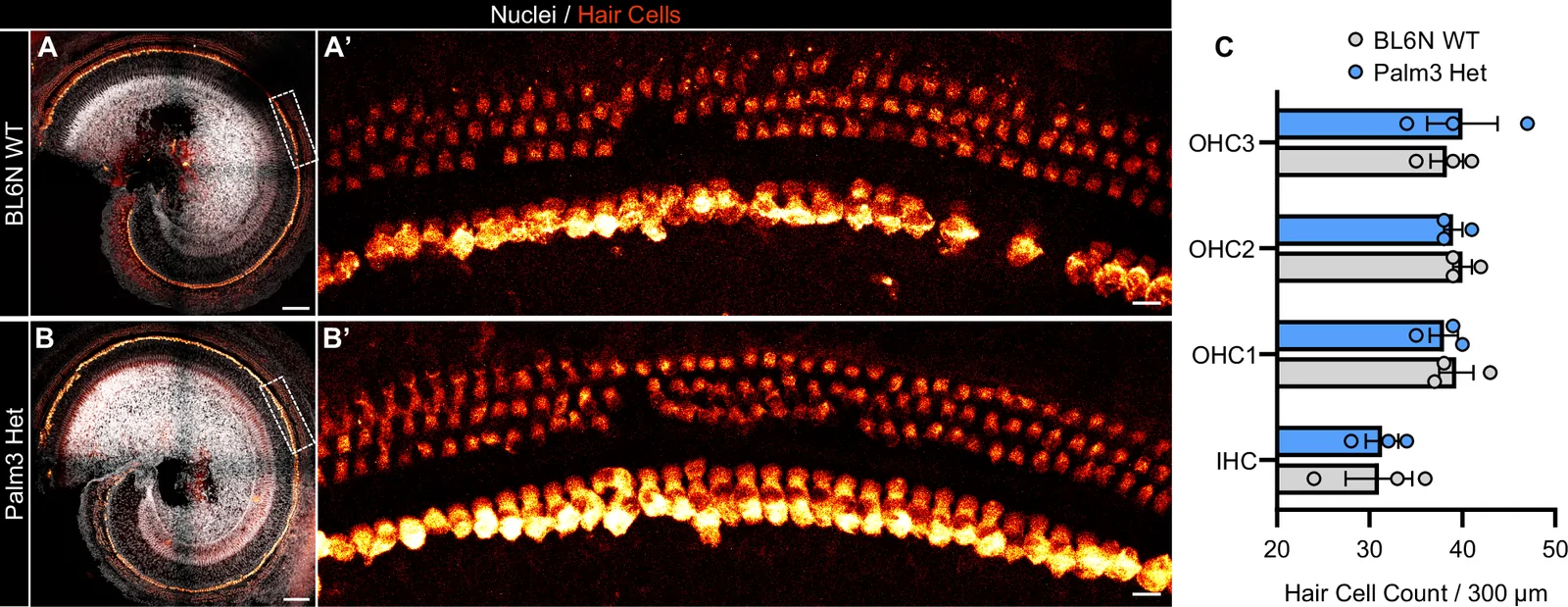
Outer and inner hair cell counts and morphology in the aged *Palm3* heterozygous mouse. **A**, **A**’, **B**, **B**’ Representative maximum projections of confocal z-stacks from organs of Corti of 1-year-old BL6N wild-type (**A**) and *Palm3* heterozygous (**B**) mice, with indicated hair cell regions magnified in **A**’, **B**’. Tissue was stained for hair cells and nuclei using Myosin7A (red) and TO-PRO-3 (gray), respectively. Scale bars: 100 µm (**A**, **B**) and 10 µm (**A**’, **B**’). **C** Bar graph showing mean ± SEM hair cell counts of inner hair cells and first, second, and third rows of outer hair cells (IHC, OHC1, OHC2, OHC3) in BL6N WT and *Palm3* heterozygous animals. *N* = 2 animals per genotype; *n* = 3 organs of Corti analyzed in total.

Consistent with these findings, a previously published *Palm3*-KO mouse exhibits auditory dysfunction with elevated ABR thresholds [2]. To assess whether reduced gene dosage might contribute to age-related vulnerability, we examined cochleae from 12-month-old heterozygous mice. Confocal imaging of the organ of Corti revealed overall preserved architecture in heterozygous mice compared with wild-type controls (Fig. 2A, B). Quantification of IHCs and OHCs showed comparable cell counts between genotypes at (Fig. 2C), suggesting that partial loss of *Palm3* does not increase age-related hair cell susceptibility. Together, these results support a potential LOF mechanism for possibility of *PALM3*-associated hearing loss.

## Discussion

Many hearing loss-associated genes that remain to be discovered are expected to be exceptionally rare and are most effectively identified through studying underrepresented populations and consanguineous families, which may harbor multiple homozygous loci [12]. While we initially suspected the proband to be diagnosed due to the homozygous *OTOA* NM_144672.4:c.1939G>C p.(Gly647Arg) variant, reanalysis uncovered the homozygous *PALM3* NM_001145028.2:c.314+1G>A variant that demonstrated aberrant splicing with two potential splice products. Exon 4 skipping would cause a frameshift (r.173_315del p.(Arg58Thrfs*47)), while skipping of exons 4–5 would cause an in-frame deletion (r.172_399del p.(Arg58_Gln133del)) removing 75 amino acid residues in the longest isoform encoding 688 amino acids (NM_001145028.2). Both outcomes are consistent with loss of normal protein function.

Although *PALM3* does not yet have an established gene-disease relationship in humans, the available evidence is insufficient to distinguish the contribution of the *PALM3* variant from the coexisting homozygous *OTOA* variant in the present family. Therefore, several interpretations remain possible, including that the *OTOA* variant alone fully explains the phenotype, that *PALM3* acts as a modifying factor influencing disease severity or age at onset, or that both variants contribute through a potential dual molecular diagnosis. Nevertheless, the observed splice defect, predicted LOF effect across all known transcripts, and concordant findings from the recently characterized *Palm3* knockout mouse model support *PALM3* as a candidate hearing loss gene warranting further investigation in additional families.

The relevance of the *Palm3* mouse phenotype is further supported by the substantial overlap between mouse and human auditory genetics. Large-scale mouse mutagenesis and phenotyping studies have identified numerous genes required for hearing, many of which were subsequently shown to cause hereditary hearing loss in humans [13–15]. More recently, systematic comparisons of mouse and human deafness genes have demonstrated extensive conservation of auditory pathways and disease mechanisms across species. Consequently, mouse models are increasingly recognized as a valuable source of candidate gene discovery, particularly for rare disorders in which human genetic evidence remains limited. In this context, the concordance between the auditory phenotype observed in *Palm3*-deficient mice and the genetic findings reported here provides additional support for *PALM3* as a candidate hearing loss gene.

The isolated hearing loss phenotype observed in the proband is consistent with a potential role for biallelic *PALM3* disruption in non-syndromic hearing loss and suggests that this gene may emerge as a DFNB-associated gene following the identification of additional families segregating biallelic *PALM3* variants. However, the observed phenotype is compatible with autosomal recessive hearing loss and may also be consistent with previously reported *OTOA* hearing loss (DFNB22). Therefore, the available clinical data do not allow clear phenotypic separation between a potential *PALM3*-related contribution and the known *OTOA* phenotype but rather highlights an important challenge in rare disease genomics, which is disentangling dual molecular diagnoses in heterogeneous disorders.

Genetic factors are estimated to account for approximately 50–70% of variability in auditory acuity in mid‑ to late‑life, underscoring the role of inherited susceptibility in ARHL [16]. Increasing evidence suggests overlap between genes implicated in congenital or early-onset deafness and those contributing to ARHL susceptibility [7,17]. Recent large-scale human genetic analyses identified *PALM3* as associated with ARHL [4], suggesting that rare functional variants may influence auditory decline across the lifespan. Three stages of hearing phenotypes were defined, including self-reported hearing difficulty, hearing difficulty in background noise, and hearing aid use. Rare variants were filtered based on minor allele frequency and predicted functional impact, such as LOF and missense changes. A burden test and Sequence Kernel Association Test – Optimal (SKAT-O) were performed to evaluate the combined impact of variants within each gene. These variant analyses identified *PALM3* associated with hearing aid use [4].

Paralemmins are membrane-associated proteins involved in membrane dynamics and cytoskeletal interactions [9]. *PALM3* is expressed in both IHCs and OHCs [18]. Disruption of membrane and cytoskeletal stability is a mechanism of hair cell vulnerability [19]. Although ABRs were not performed on the heterozygous mice, and aging to 12 months may not be old enough to model ARHL in mice, our observations, including a biallelic LOF variant in an individual affected with ARHL, aberrant splicing demonstrated in vitro, and auditory dysfunction in the *Palm3*-KO mouse, support a possible LOF effect. A recent study, highlighted reduced gene expression as sufficient to influence disease risk in genes that are sensitive to dosage changes [20].

While the contribution of the *PALM3* variant cannot currently be disentangled from the *OTOA* variant in the proband, the combined segregation, functional, and comparative model data strengthen its candidacy. These findings highlight *PALM3* as a promising candidate gene for hereditary hearing loss and a target for further functional and therapeutic studies, while underscoring the importance of continued evaluation of families harboring VUS.

## Supplementary information

Supplementary Materials

Supplementary Table 1

Supplementary Figure 1

## Data Availability

The *OTOA* and *TMC1* variants have been submitted to ClinVar under accession SUB16130109. The *PALM3*variant was submitted to ClinVar under accession SUB16231678.
