# Supplementary material for: PALM3 and hearing loss: a potential dual diagnosis interfering with novel gene discovery
Source: Eur J Hum Genet. 2026 Jul 29;34(10):1585–9. doi: 10.1038/s41431-026-02206-7 (PMC13634051; doi:10.1038/s41431-026-02206-7)
Supplement: Supplementary file 1 [file 41431_2026_2206_MOESM2_ESM.pdf]

Supplementary Table 1. Primers used for segregation testing and *in vitro* splice assay

Name	Primer sequence (5'-3')	Amplicon size (bp)
hu PALM3 Ex4-5 XhoI F	aattctcgagTCTGGGACAGTGATGCTGTT	864
hu PALM3 Ex4-5 BamHI R	attggatccTGACTTGCCAAGACCACAGA	
OTOA Ex19 F	GAATGTCTTTGGGATGAGCTCTTGG	378
OTOA Ex19 R	TTTAGAGTAAGCTTCAAGACCCACC	
TMC1 Ex 16-17 F	CCTAGTTTCTCCCTTGTGCCT	643
TMC1 Ex16-17 R	AATTCAGAGCCAGCACACAG	
SD6 F	TCTGAGTCACCTGGACAACC	NA
SA2 R	ATCTCAGTGGTATTTGTGAGC	
