# Supplementary material for: PALM3 and hearing loss: a potential dual diagnosis interfering with novel gene discovery
Source: Eur J Hum Genet. 2026 Jul 29;34(10):1585–9. doi: 10.1038/s41431-026-02206-7 (PMC13634051; doi:10.1038/s41431-026-02206-7)
Supplement: Supplementary file 2 [file 41431_2026_2206_MOESM1_ESM.docx]

**Supplementary Materials**

**Patient recruitment and clinical assessment**

We evaluated an extended consanguineous family comprising two branches with suspected different causes of hearing impairment. This family was included in a large ethnically diverse Iranian population rare disease study consisting of approximately 800 probands with the sole inclusion criteria being hereditary hearing impaired. This study was approved by the Medical Faculty of the University of Würzburg, Germany (approval numbers 131/21, 226/18 and 46/15). Blood samples were collected after obtaining informed consent from patients or their parents. Written informed consent from the parents or legal guardians of the patients/participants was obtained for the publication of their data.

Demographic, otolaryngologic, audiological, and relevant medical data were ascertained from the medical records of the proband and her husband. Affected individuals underwent a complete otologic evaluation. Routine pure-tone audiometry was performed according to current standards and measured hearing thresholds at 0.25, 0.5, 1, 2, 4, and 8 kHz. Air- and bone-conduction thresholds were measured and severity of hearing loss was determined by averaging pure-tone thresholds over 0.5, 1, 2 and 4 kHz (pure-tone average, PTA_0.5-4kHz_).

**Exome sequencing, bioinformatics, and variant classification**

The genomic DNAs of the proband and her hearing impaired partner were exome sequenced. Following extraction of DNAs from whole blood by standard protocols, proband DNA samples were subjected to exome capture using the Nextera DNA Exome (Illumina, Inc., San Diego, CA, USA) according to manufacturer’s protocols and paired-end sequenced on an Illumina sequencer. The exome data were analyzed and processed as previously described (1). Copy number variation analysis was performed. A focus on hearing loss-associated genes was first prioritized in exome analyses and later included exome-wide investigations.

The generated sequences were demultiplexed and mapped to the human genome reference (GRCh38) with Burrows Wheeler Aligner for subsequent variant calling. Bioinformatics filtering strategy focused on exonic and donor/acceptor splicing variants. Alternative alleles present at >20% and a minor allele frequency <0.01 were assessed using gnomAD (2), TopMed (3). and the All of Us research program (4). In silico pathogenicity predictions applied SIFT (5), PolyPhen-2 (6), FATHMM (7), MutationTaster (8), REVEL (9), and CADD (10). Computational assessment of splicing effects used SpliceSiteFinder-like, MaxEntScan, NNSplice, and GeneSplicer embedded in Alamut Visual Plus v1.6.1 (Sophia Genetics, Bidart, France), as well as varSEAK (JSI Medical Systems), SpliceAI Visual and AbSplice embedded in SpliceAI Visual (11). HGVS nomenclature was verified using VariantValidator (12).

Pathogenicity of identified variants in known hearing loss-associated genes was evaluated according to the hearing loss-adapted American College of Medical Genetics/Association for Molecular Pathology (ACMG/AMP) guidelines (13). Variants were analyzed in ClinVar and the Deafness Variation Database (DVD) (14) to assess previous variant classifications. Variants were subjected to segregation analysis by Sanger sequencing with the DNA samples from available family members. Primers are shown in Supplementary Table 1. Visualization of truncated variants and variant nomenclature validation was performed using Mutalyzer (15).

***In vitro* splicing assay**

RNA studies of variants were conducted following established protocols with some modifications (16, 17). In brief, the minigene construct comprised an 864 bp region containing exons 4 and 5 with flanking intronic sequence. This region was amplified from the proband and a healthy control using primers containing specific restriction sites. All primers are shown in Supplementary Table 1. The PCR fragments were ligated between exons A and B of the linearized pSPL3-vector following digestion with restriction enzymes. The recombinant vectors were transformed into DH5α competent cells (NEB 5-alpha, New England Biolabs, Frankfurt, Germany), plated and incubated overnight. After conducting colony PCR with pSPL3 Primer SD6 F and the cloning target-specific reverse primer, the results were analyzed. The sequences of both the wild-type and mutant-containing vectors were confirmed through Sanger sequencing. Subsequently, these vectors were transfected into HEK293T cells (ATCC, Manassas, VA, USA). Transfection was achieved by introducing 2 µg of the respective pSPL3 vectors using 6 µL of FuGENE 6 Transfection Reagent (Promega, Walldorf, Germany). The empty vector control was also included in the experiment as a control. The cells were then harvested 24 hours post-transfection, and total RNA was isolated using the miRNeasy Mini Kit (Qiagen, Hilden, Germany). cDNA was synthesized using the High Capacity cDNA Reverse Transcription Kit (Applied Biosystems, Waltham, MA, USA) following the manufacturer’s protocols. cDNA was PCR amplified using vector-exon specific primers. The amplified fragments were visualized on a 1% agarose gel.

**Immunohistological Analysis of Cochlear Hair Cells in *Palm3^+/−^* Mice**

*Palm3* heterozygous mice (12 months old) and wild-type mice (11 months old) were euthanized, and dissected cochleae were fixed for 1 h at 4 °C in 4% formaldehyde in PBS. Cochleae were decalcified in 0.3 M EDTA for 3 days with repeated solution changes. Apical–medial turns of the organ of Corti were then dissected for subsequent immunohistological staining (18).

Tissues were permeabilized for 30 min in permeabilization buffer (0.5% Triton X-100 in PBS) and blocked for 1 h in blocking buffer (10% goat serum in permeabilization buffer). Samples were incubated overnight at 4 °C with anti-Myosin7A antibody (MYO7A 138-1, DSHB; 1:500 dilution), followed by a 3-h incubation at room temperature with goat anti-mouse IgG1 Alexa Fluor 488 (A-21121, Invitrogen; 1:500 dilution) and TO-PRO-3 iodide (T3605, Invitrogen; 1:1000 dilution). Tissues were mounted on glass slides using a Mowiol-based mounting medium prior to imaging.

Hair cells were counted in a 300-µm-long region of interest located 1600 µm from the apex of the organ of Corti, measured along the inner hair cell row. Quantification was performed using FIJI/ImageJ.

**References**

1.	Redfield SE, De-la-Torre P, Zamani M, Wang H, Khan H, Morris T, et al. PKHD1L1, a gene involved in the stereocilia coat, causes autosomal recessive nonsyndromic hearing loss. Hum Genet. 2024;143(3):311-29.

2.	Chen S, Francioli LC, Goodrich JK, Collins RL, Kanai M, Wang Q, et al. A genomic mutational constraint map using variation in 76,156 human genomes. Nature. 2024;625(7993):92-100.

3.	Taliun D, Harris DN, Kessler MD, Carlson J, Szpiech ZA, Torres R, et al. Sequencing of 53,831 diverse genomes from the NHLBI TOPMed Program. Nature. 2021;590(7845):290-9.

4.	Genomic data in the All of Us Research Program. Nature. 2024;627(8003):340-6.

5.	Ng PC, Henikoff S. SIFT: Predicting amino acid changes that affect protein function. Nucleic Acids Res. 2003;31(13):3812-4.

6.	Adzhubei IA, Schmidt S, Peshkin L, Ramensky VE, Gerasimova A, Bork P, et al. A method and server for predicting damaging missense mutations. Nat Methods. 2010;7(4):248-9.

7.	Shihab HA, Gough J, Cooper DN, Stenson PD, Barker GL, Edwards KJ, et al. Predicting the functional, molecular, and phenotypic consequences of amino acid substitutions using hidden Markov models. Hum Mutat. 2013;34(1):57-65.

8.	Steinhaus R, Proft S, Schuelke M, Cooper DN, Schwarz JM, Seelow D. MutationTaster2021. Nucleic Acids Res. 2021;49(W1):W446-w51.

9.	Ioannidis NM, Rothstein JH, Pejaver V, Middha S, McDonnell SK, Baheti S, et al. REVEL: An Ensemble Method for Predicting the Pathogenicity of Rare Missense Variants. Am J Hum Genet. 2016;99(4):877-85.

10.	Schubach M, Maass T, Nazaretyan L, Röner S, Kircher M. CADD v1.7: using protein language models, regulatory CNNs and other nucleotide-level scores to improve genome-wide variant predictions. Nucleic Acids Res. 2024;52(D1):D1143-d54.

11.	de Sainte Agathe JM, Filser M, Isidor B, Besnard T, Gueguen P, Perrin A, et al. SpliceAI-visual: a free online tool to improve SpliceAI splicing variant interpretation. Hum Genomics. 2023;17(1):7.

12.	Freeman PJ, Hart RK, Gretton LJ, Brookes AJ, Dalgleish. VariantValidator: accurate validation, mapping, and formatting of sequence variation descriptions. Hum Mutat. 2018;39(1):61-68.

13.	Oza AM, DiStefano MT, Hemphill SE, Cushman BJ, Grant AR, Siegert RK, et al. Expert specification of the ACMG/AMP variant interpretation guidelines for genetic hearing loss. Hum Mutat. 2018;39(11):1593-613.

14.	Azaiez H, Booth KT, Ephraim SS, Crone B, Black-Ziegelbein EA, Marini RJ, et al. Genomic Landscape and Mutational Signatures of Deafness-Associated Genes. Am J Hum Genet. 2018;103(4):484-97.

15.	Lefter M, Vis JK, Vermaat M, den Dunnen JT, Taschner PEM, Laros JFJ. Mutalyzer 2: next generation HGVS nomenclature checker. Bioinformatics. 2021;37(18):2811-7.

16.	Rad A, Schade-Mann T, Gamerdinger P, Yanus GA, Schulte B, Müller M, et al. Aberrant COL11A1 splicing causes prelingual autosomal dominant nonsyndromic hearing loss in the DFNA37 locus. Hum Mutat. 2021;42(1):25-30.

17.	Tompson SW, Young TL. Assaying the Effects of Splice Site Variants by Exon Trapping in a Mammalian Cell Line. Bio Protoc. 2017;7(10).

18.	Halim VC, Bahader I, Derstroff D, Ullrich C, Kuwabara MF, Hultqvist G, et al. Paralemmin-3 sustains the integrity of the lateral plasma membrane and subsurface cisternae of auditory hair cells. bioRxiv. 2025:2025.07.11.664127.
